# Biochemical and technological properties of moose (Alces alces) recombinant chymosin

**DOI:** 10.18699/VJGB-22-31

**Published:** 2022-05

**Authors:** D.V. Balabova, A.P. Rudometov, S.V. Belenkaya, A.N . Belov, A.D. Koval, A.A. Bondar, A.Yu. Bakulina, E.A. Rukhlova, V.V. Elchaninov, D.N. Shcherbakov

**Affiliations:** Altai State University, Barnaul, Russia; State Research Center of Virology and Biotechnology “Vector”, Koltsovo, Novosibirsk Region, Russia; Altai State University, Barnaul, Russia State Research Center of Virology and Biotechnology “Vector”, Koltsovo, Novosibirsk Region, Russia Novosibirsk State University, Novosibirsk, Russia; Federal Altai Scientific Center for Agrobiotechnology, Siberian Research Institute of Cheese-Making, Barnaul, Russia; Federal Altai Scientific Center for Agrobiotechnology, Siberian Research Institute of Cheese-Making, Barnaul, Russia; Institute of Chemical Biology and Fundamental Medicine of the Siberian Branch of the Russian Academy of Sciences, Novosibirsk, Russia; State Research Center of Virology and Biotechnology “Vector”, Koltsovo, Novosibirsk Region, Russia Novosibirsk State University, Novosibirsk, Russia; State Research Center of Virology and Biotechnology “Vector”, Koltsovo, Novosibirsk Region, Russia; Federal Altai Scientific Center for Agrobiotechnology, Siberian Research Institute of Cheese-Making, Barnaul, Russia; Altai State University, Barnaul, Russia State Research Center of Virology and Biotechnology “Vector”, Koltsovo, Novosibirsk Region, Russia

**Keywords:** moose, recombinant chymosin, milk-clotting activity, biochemical properties, cheese-making, Alces alces, лось, рекомбинантный химозин, молокосвертывающая активность, биохимические свойства, сыроделие, Alces alces

## Abstract

Recombinant chymosins (rСhns) of the cow and the camel are currently considered as standard milk coagulants for cheese-making. The search for a new type of milk-clotting enzymes that may exist in nature and can surpass the existing “cheese-making” standards is an urgent biotechnological task. Within this study, we for the first time constructed an expression vector allowing production of a recombinant analog of moose chymosin in the expression system of Escherichia coli (strain SHuffle express). We built a model of the spatial structure of moose chymosin and compared the topography of positive and negative surface charges with the correspondent structures of cow and camel chymosins. We found that the distribution of charges on the surface of moose chymosin has common features with that of cow and camel chymosins. However, the moose enzyme carries a unique positively charged patch, which is likely to affect its interaction with the substrate. Biochemical and technological properties of the moose rChn were studied. Commercial rСhns of cow and camel were used as comparison enzymes. In some technological parameters, the moose rChn proved to be superior to the reference enzymes. Сompared with the cow and camel rСhns, the moose chymosin specific activity is less dependent on the changes in CaCl2 concentration in the range of 1–5 mM and pH in the range of 6–7, which is an attractive technological property. The total proteolytic activity of the moose rСhn occupies an intermediate position between the rСhns of cow and camel. The combination of biochemical and technological properties of the moose rСhn argues for further study of this enzyme.

## Abstract (ru)

Эталонными коагулянтами молока для сыроделия в настоящее время считаются рекомбинантные химозины (рХн) коровы и верблюда. Нахождение молокосвертывающих ферментов, способных превзойти эталонные коагулянты молока, является актуальной биотехнологической задачей. Нами сконструирован экспрессирующий вектор, который позволил впервые получить рекомбинантный аналог химозина лося в системе экспрессии Escherichia coli (штамм SHuffle Express). Построена модель пространственной структуры химозина лося, и проведено сравнение топографии положительных и отрицательных поверхностных зарядов с соответствующими структурами химозинов коровы и верблюда. Обнаружено, что распределение зарядов на поверхности химозина лося имеет общие черты с распределением зарядов химозинов коровы и верблюда. Отличительная особенность химозина лося – наличие положительно заряженного поверхностного участка, который, вероятно, влияет на его взаимодействие с субстратом. Исследованы основные биохимические и технологические свойства рХн лося. В качестве ферментов сравнения использовали коммерческие рХн коровы и верблюда. Установлено, что по некоторым технологическим показателям рХн лося превосходил ферменты сравнения. По сравнению с рХн коровы и верблюда специфическая активность рХн лося в меньшей степени зависит от изменения концентрации CaCl2 в диапазоне 1–5 мМ и рН в диапазоне 6–7, что является привлекательным технологическим свойством. По общей протеолитической активности рХн лося занимает промежуточное положение между ферментами коровы и верблюда. Совокупность биохимических и технологических свойств рХн лося свидетельствует о необходимости дальнейшего изучения этого фермента.

## Introduction

The segment of recombinant enzymes occupies a significant part of the modern biotechnology market (Trono, 2019). One of the first industrial enzymes obtained using genetic engineering technologies was cow’s recombinant chymosin (Flamm, 1991), which has been considered the standard of a milk-clotting enzyme (ME) in cheese-making for a long time (Belov et al., 2009; Jacob et al., 2011). The rapid development of molecular biology methods (primarily next-generation sequencing and genetic engineering) has intensified the search for enzymes with superior biochemical and technological properties compared to the milk coagulants traditionally used in the industry.

The main goal of such a search is to find the enzymes possessing approximately the same sensitivity to pH and concentration of calcium ions in milk compared with a bovine (Bos taurus) chymosin but would outperform it in a milkclotting activity (MA) and, at the same time, would demonstrate a lower overall proteolytic activity (PA) and thermal stability (TS).

Previously, researchers have obtained and studied rChns of sheep (Ovis aries) (Rogelj et al., 2001), goat (Capra hircus) (Vega-Hernández et al., 2004; Vallejo et al., 2012; Tyagi et al., 2016), water buffalo (Bubalus arnee bubalis) (Vallejo et al., 2012; Tyagi et al., 2017), and camel (Camelus dromedarius) (Kappeler et al., 2006). It was shown that the rChns of goat, buffalo, and sheep are ordinary ME and cannot compete with the bovine Chn. The camel rChn showed a higher affinity toward bovine κ-casein (κ-Cs) and had a better MA/PA ratio than the cow rChn, but was inferior to the bovine enzyme in TS (Bansal et al., 2009). Nevertheless, after a comprehensive study of its biochemical and technological properties, the camel’s rChn is widely used in the practice of cheese-making (Bansal et al., 2009; Moynihan et al., 2014; Gumus, Hayaloglu, 2019) and is now considered a reference ME along with the bovine rChn.

Later, the rChns of yak (Bos grunniens) (Luo et al., 2016; Ersöz, İnan, 2019), alpaca (Vicugna pacos) (Belenkaya et al., 2018), and Altai maral (Cervus elaphus sibiricus) (Belenkaya et al., 2020a, b) have been obtained and studied. The complete biochemical and technological characteristics of the yak rChn have not yet been established. The available literature data indicate, on the one hand, that the yak rChn has a low technologically significant threshold for TS, and on the other hand, a higher total PA, compared to that of the bovine rChn (Belenkaya et al., 2020с). According to our data, the rChn of maral has an excessively high total PA and TS, limiting its potential use only to the production of cheeses with short ripening and storage periods (Belenkaya et al., 2020a). The genetically engineered Chn of alpaca surpasses the bovine rChn in the MA/PA ratio but is inferior in this parameter to the rChn of the camel. In addition, similar to the rChn of camel, the rChn of alpaca has a higher TS than the bovine rChn. Taken together, the facts mentioned above show that despite the presence of some interesting characteristics, the complex of biochemical and technological properties of the rChns of yak, alpaca, and maral hardly allows these enzymes to be considered as an alternative to the rChns of cow and camel. 

Here we present a new milk-clotting enzyme – recombinant chymosin of moose (rChn-Alc) in a prokaryotic expression system and investigate some of its biochemical properties in comparison with standard milk-clotting enzymes. The recombinant prochymosin (rProChn) of moose developed in the prokaryotic expression system was activated by a stepwise pH change method, and an active rChn-Alc preparation capable of effectively coagulating cow’s milk was obtained. It was shown that compared to the reference milk coagulants, the specific enzymatic activity of rChn-Alc was less sensitive to changes in the H+ concentration in the pH range of 6.0–7.0. Concerning an important technological indicator, the total PA, rChn-Alc was found to occupy an intermediate position between the cow rChn and the camel rChn. The specific MA of the moose rChn was lower than that of the cow and camel rChns, possibly due to incomplete refolding of the enzyme obtained in the E. coli expression system. The results obtained expand the understanding of the biochemical and technological properties of Chns of various species and create a basis for further search for technological coagulants of cow’s milk that would surpass the existing reference milk-converting enzymes in their properties

## Materials and methods

Work organization. The optimization of the structure of the moose prochymosin gene and the construction of a producer strain were carried out at the State Research Center of Virology and Biotechnology “Vector”. Works on obtaining a preparation of recombinant moose prochymosin and determining its biochemical and technological properties were carried out at Altai State University. All work was carried out in 2019.

Strains and media. Escherichia coli strain NEB Stable used to construct and propagate all plasmids was purchased from New England Biolabs (NEB, Ipswich, USA). E. coli strain SHuffle express was purchased from New England Biolabs (NEB, Ipswich, USA) and used as a heterologous host to produce the rProChn of moose (GenBank MT542132). The medium Lysogeny broth (LB) (1.0 % bacto-peptone, 0.5 % yeast extract, and 1.0 % NaCl) in liquid or solid (1.5 % agar) form was used to culture NEB stable cells at 37 °C. E. coli SHuffle express cells were cultured at 30 °C in LB medium (AppliChem, USA) with the addition of isopropyl-β-D-1- thiogalactopyranoside (IPTG) for induction (final concentration 1.0 mM).

Subcloning of prochymosin gene into pET21а expression vector. Codon optimization of the moose prochymosin sequence (accession number MT542132) for the selected expression system was performed by the online service Integrated DNA Technologies (https://eu.idtdna.com/CodonOpt), followed by synthesis and integration into pGH cloning plasmid. Synthetic gene sequence containing BamHI and HindIII restriction sites at the 5′- and 3′-ends, respectively, was digested and subcloned into the expression vector pET21a (Novagen, Germany). The structure of the recombinant plasmid was verified by Sanger sequencing. As a result, the expression vector pET21-CYM-Alc was obtained.

E. coli transformation and recombinant protein production. For obtaining the target protein, the chemical transformation of E. coli strain SHuffle express was carried out with the resulting construct. Individual E. coli colonies containing recombinant plasmids were cultured overnight on an orbital shaker (Biosan, Latvia) in LB medium containing 100 μg/ml ampicillin at 37 °C and 180 rpm. The inoculum in a ratio of 1/100 was transferred to an Erlenmeyer flask containing LB medium and grown at 37 °C and 180 rpm. After the optical density (OD600) reached a value of 0.8, IPTG (Anatrace Products, USA) was added to the mixture to a final concentration of 1.0 mM. The culture was additionally incubated on a shaker for 12 h at 25 °C and 180 rpm. The biomass was centrifuged for 20 min at 5000 g and 4 °С to precipitate the inclusion bodies. E. coli cells were then resuspended in STET buffer (AppliChem, USA) (8.0 % sucrose; 50 mM Tris-HCl; 20 mM EDTA; 5.0 % (w/v) Triton X-100, pH 8.0) in proportion of 20 ml per 1 gram of biomass and incubated overnight at 4 °C. Thereafter cells were destroyed using a Soniprep 150 Plus ultrasonic homogenizer (MSE, PRC). Inclusion bodies were precipitated by centrifugation at 20,000 g for 20 min at 4 °C (Wei et al., 1999). The sedimented inclusion bodies were solubilized in buffer A (50 mM KH2PO4, 150 mM NaCl, pH 10.7) containing 8.0 M urea, incubated for 24 h at 15 °C and centrifuged at 20,000 g for 20 min.

Further work was carried out with a supernatant containing recombinant ProChn (rProChn). The target protein was renaturated according to the method of Wei et al. (1999). The supernatant was diluted 3× with buffer A and incubated for 12 h at 15 °C. Following the incubation, the supernatant diluted with alkaline buffer was adjusted to pH 8.0 with 1.0 M HCl, kept at 15 °C for 1 h, and dialyzed against buffer B (50 mM Tris, 150 mM NaCl, pH 8.0) overnight at 4 °C (Wei et al., 1999). As a result, an experimental preparation of moose rProChn was obtained.

The recombinant protein production in E. coli cells was analyzed by sodium dodecyl sulfate polyacrylamide gel electrophoresis (SDS-PAGE) according to the Laemmli method (Laemmli, 1970). To analyze the electrophoretic mobility of the protein and determine molecular weights, the molecular weight markers PageRuler Unstained Protein Ladder (Thermo Scientific, USA) were used. Protein concentration was measured by the Bradford method (Bradford, 1976).

Activation of moose recombinant prochymosin. Activation of the moose rProChn was carried out by a stepwise changing of pH (Belenkaya et al., 2020b). To avoid autocatalytic conversion (Pedersen et al., 1979) of zymogen to an active enzyme after the isolation from inclusion bodies and partial purification, the preparations of moose rProChn were stored in weakly alkaline conditions (buffer B) prior to activation. For activation, HCl (2.0 M) was added to the rProChn sample, adjusting pH to 3.0 with continuous stirring. Then stirring was stopped, and the mixture was incubated at pH 3.0 for 2 h. After incubation, pH of the sample was adjusted to 5.8 using 0.5 M NaOH. As a result, the rChn of moose (rChn-Alc) was obtained.

Comparison preparations of commercial reference coagulants. Biochemical properties of rChn-Alc were compared with the properties of commercial reference coagulants: bovine rChn (rChn-Bos) (granular dry form with declared MA – 2201 IMCU/g) and commercial camel rChn (rChn- Cam) (liquid form with declared MA – 1000 IMCU/ml), produced by “Chr. Hansen” (Denmark).

Three-dimensional structure modeling and imaging. The homology model of the moose Chn three-dimensional structure was built on Swiss-model server (Waterhouse et al., 2018). The bovine Chn structure was used as a template for the modeling (Jensen et al., 2013). The images of surface charges were built with Coulombic Surface Coloring function of Chimera 1.14 software package

Milk-clotting activity assays. A 10.0 % solution of standardized skimmed milk powder (MZSF OJSC, Russia) in 5 mM CaCl2, pH 6.5, was used as a substrate. A 0.5 % aqueous solution of a dry bovine rChn with a certified MA value was used as a control. Prior to determining the MA, the control sample and the liquid preparation of rChn were kept in a water bath at 35 °C for 15 min and cooled to room temperature. The procedure for determining MA was carried out in a water bath at 35 °C. Substrate solution (2.5 ml) was placed into a glass tube and heated at 35 °C for 5 min. An aliquot (0.25 ml) of an enzyme was added to the substrate, a stopwatch was turned on, and the resulting reaction mixture was immediately thoroughly mixed. The time when the first flakes of the coagulated substrate were observed in the drops of reaction mixture applied onto the tube wall was considered to be the clotting time. The milk-clotting activity was expressed in arbitrary units (AU) per 1 ml (AU/ml) and calculated using the equation:

**Formula1.**
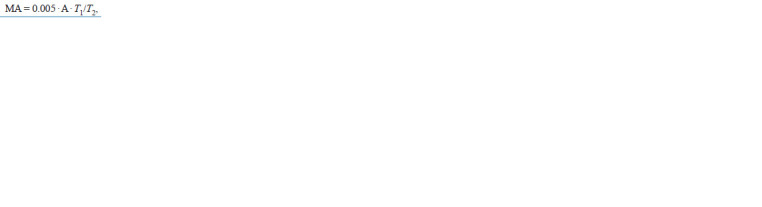
Formula-1

where A – certified MA value of the control rChn sample in AU per 1 gram, 0.005 – the dilution factor, T1 – coagulation time for the control rChn sample of chymosin, T2 – coagulation time for the test rChn sample.

Determination of total MA in each sample was performed in triplicate (n = 3). The relative MA of rChn-Alc was calculated after determining the total MA and protein concentration. For determining the relative MA of commercial reference chymosins, a 1.0 % aqueous solution of rChn-Bos was prepared, and the liquid rChn-Cam was diluted 10 times with distilled water. Protein concentration was determined in the resulting solutions with the Bradford assay. The MA values declared by the manufacturer were used to calculate the relative MA of the commercial reference enzymes. The relative MA was expressed in AU per milligram (AU/mg). To convert IMCU (International Milk Clotting Units) values into AU, a multiplication coefficient of 125 was used.

General proteolytic activity assays. A 1.0 % solution of Hammerstein-grade casein in a 20 mM Na-phosphate buffer (pH 5.6) was used as a substrate. The investigated MEs were introduced into the substrate solution in a 1:4 ratio and incubated at 35 °C for 0 (‘zero’ point), 30, 90, and 180 min. The reaction was stopped by adding trichloroacetic acid. The precipitates were filtered, and the OD of the filtrate was measured at 280 nm (OD280) with a ‘zero’ point as a control. To assess the specificity of the rChn preparations, the OD280 values of the samples incubated for 180 min were designated as the PA values. The specificity was defined as the ratio of MA to general PA (MA/PA). When calculating the specificity of rChn-Bos and rChn-Cam, the MA values stated by the manufacturer were used. The enzymes studied were normalized by МA.

Thermal stability assays. Aliquots of ME were heated in the temperature range of 30–60 °C for 30 min and then assessed for residual MA. The MA values obtained in the samples heated at 30 °C were assigned as 100 %. The enzymes studied were normalized by МA.

Dependence of rennet coagulation time on pH. Solutions (10.0 %) of standardized skimmed milk (SSM) were adjusted to pH levels of 6.0, 6.2, 6.4, 6.6, 6.8 and 7.0, and the rennet coagulation time (RCT) of the studied preparations of rChns was then determined. The RCT values at a pH of 6.0 was assigned as 100 %. The enzymes studied were normalized by МA.

Dependence of rennet coagulation time on the calcium chloride concentration. Dry powder of CaCl2 was added to the SSM to a final concentration of 1–5 mM, and the clot formation time was measured therein. The RCT values obtained in CaCl2-free samples of SSM were taken as 1.0. The enzymes studied were normalized by МA.

## Results

**Fig. 1.**
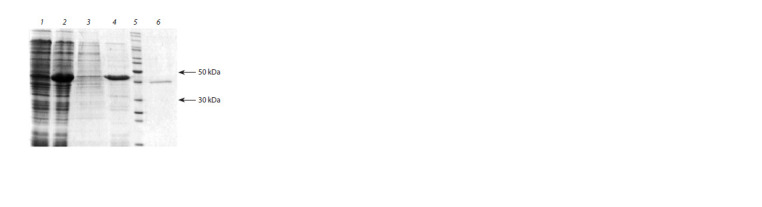
SDS-PAGE analysis of protein samples obtained from the producer strain cells: 1 – producer cell biomass before IPTG adding; 2 – producer cell biomass after 5 h IPTG adding; 3 – soluble biomass fraction after treatment with STET buffer; 4 – insoluble fraction (inclusion bodies) after treatment with lysis buffer; 5 – molecular-weight markers (200, 150, 120, 100, 85, 70, 60, 50, 40, 30, 25, 20 and 15 kDa); 6 – moose rChn obtained as a result of zymogen activation.

Expression of recombinant moose prochymosin. We used the E. coli strain SHuffle express to produce moose prochymosin in the laboratory to study its biochemical properties since E. coli is the most studied system for the expression of heterologous genes; functionally active chymosins of a number of mammals have already been obtained in this system (Rogelj et al., 2001; Belenkaya et al., 2020a, b), and despite the presence of drawbacks it allows obtaining samples of recombinant proteins in quantities sufficient for primary biochemical analysis in a short time. To obtain a producer strain, the designed nucleotide sequence of the moose ProChn gene, 1095 bp in size, was synthesized and cloned as part of the pET21a plasmid vector. The production and purification of the target protein were carried out as described previously (Belenkaya et al., 2018, 2020a). In order to evaluate the efficiency of the synthesis of rProChn of moose, as well as to determine its localization in the E. coli cell, an electrophoretic analysis of protein preparations obtained from the cells of the producer strain was carried out (Fig. 1).

Analysis of E. coli cells containing the pET21-CYM-Alc plasmid after induction with IPTG showed a high protein content, which coincides with the calculated one for rProChn of moose in terms of electrophoretic mobility (41 kDa). Its content was ≥30 % (see Fig. 1, lane 1) of the total amount of cell proteins. It can be seen that the soluble fraction of E. coli biomass after treatment with STET buffer and centrifugation (lane 3) contains almost no target protein, while the fraction of inclusion bodies is nearly completely represented by rProChn of moose (lane 4).

Activation of rProChn and obtaining of rChn-Alc. The initial MA of the rProChn was <1.0 AU/ml. After activation, MA was equal to 843 AU/ml. Thus, as a result of activation, the total MA of the preparation increased more than 840 times, indicating the efficiency of the conversion of rProChn into active rChn of moose. In this case, a propetide is cleaved from the N-terninus of prochymosin molecule, resulting in a change in the length of the protein in the polyacrylamide gel, which is recorded using SDS-PAGE analysis (see Fig. 1, lane 6).

Three-dimensional structure and surface charges of chymosin. Analyses of Chn sequences from different mammals demonstrated that the moose Chn is close to the other ones, especially the bovine Chn. Amino acid sequences of the bovine and moose Chn share 93.5 % identity, differing in 21 out of 323 positions. Three-dimensional structures of proteins with such a high similarity level are expected to be very close. In comparison, the camel and bovine Chn have 83.3 % identity, and their structures are similar. Therefore, we built a homology model of the moose Chn and used it for analyzing the surface charges.

**Fig. 2.**
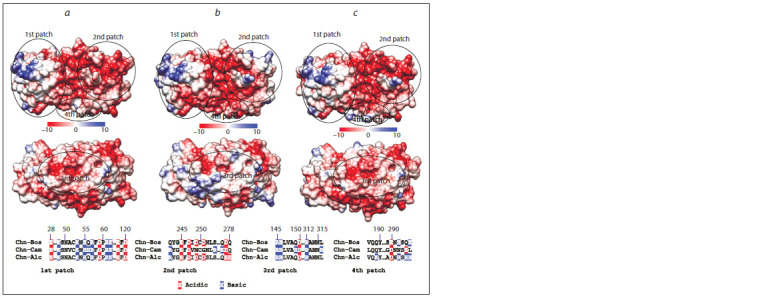
Surface charged patches on chymosin: bovine (a), camel (b), moose (c). Molecular surfaces are colored with Chimera software by the potential values in kcal/mole at 298 K. All proteins oriented with the C-terminal domain to the left and the N-terminal domain to the right, looking into the binding cleft (top) and rotated 180 degrees around the horizontal direction (bottom). The sequences of the chymosin charged patches are aligned, charged residues are highlighted.

Previous studies of camel and bovine chymosins revealed three positively charged patches on their surfaces that can contribute to the enzyme-substrate interaction (Jensen et al., 2013). Patch 1 and patch 3 are identical in the bovine and moose Chn, and patch 2 in the moose Chn has the same total charge as patch 2 in the bovine Chn, but charge distributions are different. Also, an additional charged patch in the moose Chn can be seen, designated as patch 4 (Fig. 2).

## Technological properties

Specific MA. Milk-clotting activity is a basic technological characteristic of any new rChn since it indicates its ability to hydrolyze the Chn-sensitive peptide bond in the kappa-casein molecule and cause milk coagulation. Specialists in cheesemaking are aware of the paradox “cow Chn – camel milk and camel Chn – cow milk”. The paradox lies in the inability of the cow Chn to coagulate camel milk, while the camel enzyme effectively coagulates cow milk (Kappeler et al., 2006). Therefore, the study of any new ME for cheese-making begins with determining its MA in relation to cow’s milk as the main raw material for cheese production. Only when one is sure that the new enzyme is capable of coagulating cow’s milk is it reasonable to start investigating its other technological properties. Since the cow rChn and the camel rChn can be considered reference MEs for cheese-making, it is advisable to compare the biochemical properties of the new milk coagulant with them in order to assess its technological prospects.

In terms of specific MA, the moose rChn was inferior to the reference MEs – the cow and camel rChns – by 2.7 and 4.4 times, respectively (Table 1). The specific MA of rChn- Cam is 1.61 times higher than the specific coagulation activity of rChn-Bos. This is in good agreement with the data of (Kappeler et al., 2006; Belenkaya et al., 2020c), where it was shown that the ratio of the specific MA of rChn-Bos to the rChn-Cam is 1:1.7

**Table 1.**
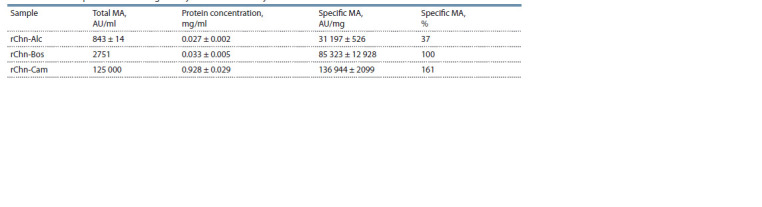
Total and specific milk-clotting activity of recombinant chymosins

General proteolytic activity and specificity. To predict the technological prospects of any new ME, it is necessary to study its general PA. Excessive PA of milk coagulant is considered a negative factor in cheese production since it leads to a decrease in the yield and deterioration of the organoleptic properties of the cheeses produced (Singh et al., 2003; Harboe et al., 2010).

Conventionally, the PA of milk coagulants can be divided into specific and non-specific. The specific or milk-clotting activity of ME provides the hydrolysis of the F105-M106 bond in the κ-casein molecule, causing the destabilization of casein micelles and leading to the formation of a milk clot. Non-specific or general PA characterizes the ability of ME to hydrolyze any peptide bonds, with the exception of the F105-M106 bond of κ-casein. The ideal milk coagulant for cheese-making should exhibit the maximal MA with the minimal general PA (Harboe et al., 2010). The ratio of MA to general PA (MA/PA) is called specificity. The higher the value, the more versatile the ME, and the wider the range of cheeses to be produced.

**Fig. 3.**
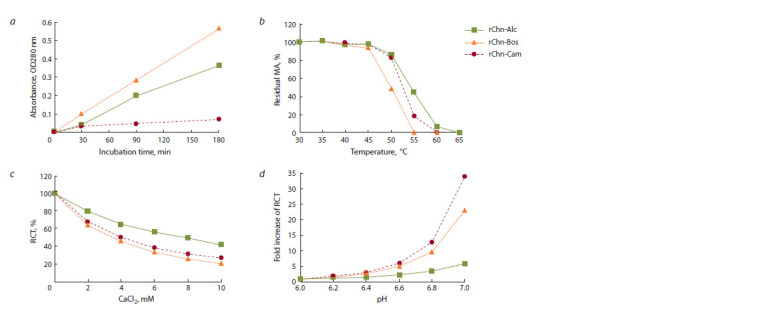
Results of a comparative study of the following dependency patterns: general proteolytic activity (OD280) on the incubation time (a), residual MA (%) on the heating temperature (b), RCT on the calcium chloride concentration (c) and pH (d).

The dynamics of accumulation of milk substrate proteolysis products under the action of rChn-Alc is similar to rChn-Bos and differs markedly from rChn-Cam (Fig. 3, a). These differences are most clearly observed after 90 min of incubation. After 180 min of incubation of the enzyme-substrate mixture, the general PA values (expressed in OD280 units) of the rChns of moose, cow, and camel were 0.362 ± 0.023, 0.565 ± 0.020, and 0.072 ± 0.012, respectively. As expected, rChn-Cam showed an exceptionally low level of non-specific proteolysis, which is in good agreement with the data (Bansal et al., 2009), according to which the PA of rChn-Cam was 4 times lower than that of rChn-Bos. Apparently, low values of the general PA are typical for the rChns of representatives of the Camelidae family. According to our data, the general PA of another member of this family, the alpaca, is about 3 times lower than that of the cow (Belenkaya et al., 2018).

If we take the general PA of rChn-Bos as 100 %, then the PA of rChn-Alc and rChn-Cam will be 64 and 13 %, respectively. Using data on specific MA and general PA, specificity can be calculated (Table 2).

**Table 2.**
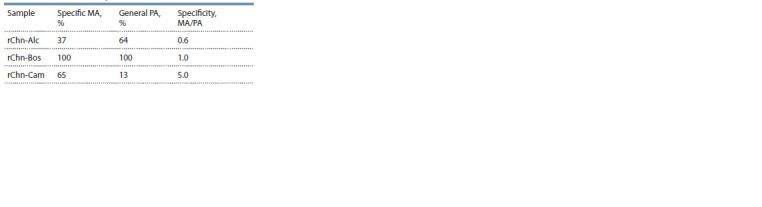
Specific MA, general PA, and specificity of recombinant moose, cow, and camel chymosins

By specificity, and hence by the degree of cheese-making universality, the studied enzymes were arranged in the following order: rChn-Cam > rChn-Bos > rChn-Alc. The ratio of MA/PA calculated for the moose rChn was 1.6 and 8.3 times lower than for the cow and camel rChns. It is possible that the low specificity of rChn-Alc is a consequence of its low specific MA, which, as we have already noted, may be due to incomplete refolding of its zymogen. On average, the efficiency of restoring the correct folding (in terms of MA) of genetically engineered chymosins obtained after solubiliza- tion of inclusion bodies rarely exceeds 30 % (Wei et al., 1999, 2000; Chen et al., 2000; Eskandari et al., 2012).

Thermostability. Milk coagulants with a high threshold of thermal inactivation may show undesirable PA at the stages of cheese production associated with an increase in the heating temperature of the clot, as well as during prolonged maturation and storage of finished products. Therefore, the TS is an important technological characteristic of any new ME that claims to be used in cheese-making.

The proteolytic activity of MEs is registered in various types of cheeses (Masotti et al., 2010; Sforza et al., 2012; Gumus, Hayaloglu, 2019; Lamichhane et al., 2019; D’Incecco et al., 2020; Mane, McSweeney, 2020) and makes a significant contribution to the “proteolytic maturation” of the product. Information about the TS of the milk coagulant used allows one to regulate the degree of proteolysis and influence the maturation time of cheeses by varying the processing temperature of the cheese grain or by using ME with different thermal activation thresholds (Lamichhane et al., 2019).

It was found that the rChn of a camel is more thermally stable than that of a cow (Kappeler et al., 2006; Jensen et al., 2013; Belenkaya et al., 2020с). It is also known that the general PA of these enzymes increases with increasing temperature – rChn-Bos shows the maximum PA at 55.0 °C, and rChn-Bos at 52.5 °C (Kappeler et al., 2006). An increase in the heating temperature of the clot from 50 to 56 °C when producing very hard, granular, cows’ milk cheese using the rChn of a cow or a camel leads to a significant decrease in the concentration of products of proteolysis of αS1-casein. But even after processing the clot at 56 °C, the concentration of markers of proteolysis of αS1-casein was higher in maturing and stored cheeses produced using a more thermally stable rChn-Cam than when using rChn-Bos (Costabel et al., 2015). This is despite the fact that the general PA of a camel enzyme is 3.5–4.0 times lower than that of a cow (Kappeler et al., 2006).

The ranges of TS of the rChns of the same species obtained in different expression systems may differ. The thresholds of total temperature inactivation of the camel rChn expressed in higher mold fungi (Aspergillus niger) and yeast (Komagataella (Pichia) pastoris) differed by 10 °C (Belenkaya et al., 2020с). The experimental rChn-Bos synthesized in the E. coli BL21(DE3) system exceeded the commercial rChn-Bos expressed in A. niger by 15 °C (Belenkaya et al., 2018). These data indicate a possible role of posttranslational modifications as a factor influencing the temperature stability of rChns

The threshold of thermal inactivation was considered the T (°С) at which the studied rChn lost >20 % of the initial coagulation activity. According to this criterion, the TS threshold for rChn-Bos was 50 °С, and for rChn-Alc and rChn-Cam – 55 °С (see Fig. 3, b). After 30 min of heating up at 55 °С, rChn-Bos was completely inactivated. Despite the same TS threshold, rChn-Alc and rChn-Cam differed in the dynamics of thermal inactivation in the temperature range of 50–65 °C. After heating up to 55 °C, the residual coagulation activity of the moose rChn was almost 2.5 times higher than that of rChn-Cam and amounted to 44.9 and 18.2 %, respectively. The recombinant camel rChn was completely inactivated after heating at 60 °C, while rChn-Alc still retained 6.5 % of the original MA at this temperature, suggesting higher temperature stability of rChn-Alc compared to rChn-Cam.

Thus, taking into account the same threshold of thermal inactivation of rChn-Alc and rChn-Cam, according to the TS criterion, the studied enzymes are arranged as follows: rChn-Alc > rChn-Cam > rChn-Bos. Increased, in comparison with reference enzymes, TS limits the scope of application of the moose rChn assumes its use, first of all, in the production of cheeses with short maturation and storage periods

Dependence of rennet coagulation time on the calcium chloride concentration. Most rennet cheeses are made from pasteurized milk. It is known that during pasteurization, denatured β-lactoglobulin binds to micellar κ-casein, which leads to an increase in the duration of RCT (Fox et al., 2017). In addition, during high-temperature processing of raw milk, part of the salts and calcium ions present in it precipitates in the form of insoluble calcium phosphate. As a result, the concentration of Ca2+ in milk decreases, which also increases the RCT. In order to avoid increasing the dose of introduced ME and improve the coagulation ability of pasteurized milk, CaCl2 is added to it in an amount of 0.1–0.4 g/l (~1–4 mM). However, an increase of the CaCl2 concentration in the milk substrate causes not only an increase in the coagulation activity but also in the general PA of the enzyme, especially at the stage of milk coagulation (Wang et al., 2015). Therefore, the use of ME with high sensitivity to Ca2+ concentration is associated with the risk of negative consequences of increasing its general PA. Based on this, it is necessary that the new milk coagulant, in comparison with modern reference technological enzymes, has a comparable or lower sensitivity to changes in the concentration of CaCl2 in the milk substrate.

Just as in the case of other MEs (Fox et al., 2017), the duration of RCT under the action of the studied rChns decreased in response to an increase in the concentration of calcium chloride. In the range of 0–10 mM of CaCl2 clot formation time is reduced by 0–58 % for rChn-Alc, 0–79 % for rChn- Bos, and 0–73 % for rChn-Cam. The dynamics of changes in the dependence of RCT on the concentration of calcium chloride for the cow and camel rChns is almost the same (see Fig. 3, c). Recombinant Chn of moose differs from reference enzymes – its coagulation activity is less sensitive to changes in the concentration of CaCl2 in the milk substrate. At 4 mM CaCl2, the RCT of the milk substrate decreases by 2.0 and 2.2 times, respectively, under the action of rChn-Bos and rChn- Cam, and only by 1.5 times for rChn-Alc. This, in particular, means that the risk of an increase in the general PA when using the moose rChn to curdle pasteurized milk with added CaCl2 is much less than that of reference coagulants, which is a positive factor from the point of view of cheese production.

Dependence of rennet coagulation time on pH. The optimums of the specific activity of various types of chymosins lie in the pH range of 4.6–6.0 (Belenkaya et al., 2020с). However, in the production of most types of rennet cheeses, ME is added to the milk mixture at a pH of 6.5–6.6. Therefore, one of the technological requirements for any new coagulant is its ability to effectively curdle milk in a slightly acidic pH range that is far from the pH optimum.

The duration of RCT depends on the electrostatic and hydrophobic properties of casein micelles, which are related to the H+ concentration. When milk is acidified, the total negative charge of caseins decreases due to the pH approaching the pI values. This reduces the forces of electrostatic repulsion between the micelles and simultaneously increases the casein-casein hydrophobic interactions, which accelerates the formation of milk clot. If the pH increases, the casein-casein hydrophobic interactions weaken as the total negative charges of caseins increase. The growing forces of electrostatic repulsion prevent the convergence of similarly charged casein micelles and slow down the formation of milk clot (Lucey, 2002; Harboe et al., 2010; Fox et al., 2017).

By the nature of the dependence of the coagulation ability on pH, the most promising for cheese-making are MEs, which slowly lose activity when moving away from the pH-optimum to the alkaline region and can exhibit high MA at weakly acidic and neutral pH values.

Compared to the reference rChns, the coagulation activity of rChn-Alc is much less dependent on changes in milk pH from 6.0 to 7.0 (see Fig. 3, d ). At a pH of 6.4–6.6, the RCT for rChn-Alc increases 1.6–2.3 times, and for rChn-Bos and rChn-Cam, this parameter increases 2.5–5.0 and 2.9–6.0 times, respectively. At pH 7.0, the rChns of cow and camel showed extremely low (we can say, trace) coagulation activity, and the differences between them and the rChn of moose were most clearly manifested. Based on the data obtained, it can be argued that in the working “cheese-making” pH range of 6.5–6.6, the consumption of the moose rChn will be lower than that of the reference MEs, which is an important technological characteristic. However, the ability of rChn-Alc to show significant coagulation activity at neutral pH values is not unique. Previously, similar properties were found in the rChns of the goat (Vallejo et al., 2012) and yak (Ersöz, İnan, 2019).

Thus, the moose rChn is able to effectively curdle cow’s milk at a pH of 6.5–6.6, and is not inferior in this indicator to commercial genetically engineered chymosins.

## Discussion

For the first time, recombinant moose chymosin was obtained, and its characteristics, important for the production of rennet cheeses, were also investigated. We have chosen a prokaryotic expression system for preliminary characterization of the enzyme, because it is easier to work with and since it was known that other recombinant chymosins obtained in prokaryotes retain their activity (Eskandari et al., 2012; Belenkaya et al., 2020a, c). The conditions used for the expression of the moose chymosin gene in the E. coli system lead to a highly efficient synthesis of the target protein, with almost all of it accumulating in an insoluble form in inclusion bodies. As expected, МА rProChn was very low, and after activation MA rChn-Alc it was 843 AU/ml. According to the total MA, the moose rChn obtained by us was 2.4–2.8 times inferior to other genetically engineered rChns (2014 AU/ml for alpaca and 2330 AU/ml for maral) obtained in the E. coli expression system (Belenkaya et al., 2018, 2020a, b).

It is possible that the low specific MA of rChn-Alc, compared with the reference milk coagulants, is due to the insufficient efficiency of its zymogen refolding after isolation from the inclusion bodies. It is known that the stage of restoring the correct three-dimensional structure is a “bottleneck” in obtaining rChn in E. coli expression systems and leads to a decrease in the yield and specific activity of the target product (Wei et al., 1999, 2000; Chen et al., 2000; Eskandari et al., 2012).

Also, we cannot exclude the possibility that under our conditions the moose chymosin was not activated quite correctly, with the N-end cut off in a different position, thus affecting its activity. For example, loss of the first three residues of camel chymosin significantly decreased its activity (Jensen et al., 2013). We have so far characterized only the enzymatic properties and determined the approximate molecular weight using SDS-PAGE analysis, but we do not know the exact amino acid sequence of rChn-Alc.

Previously, it was suggested that the technological properties of the camel Chn depend on its surface charge distribution (Jensen et al., 2013). The total charge of κ-casein C-terminal part is negative, as is the total charge of all known chymosins. Positively charged patches on the chymosin surface can play a role in properly positioning and binding the enzyme to the substrate (Jensen et al., 2013). Most positively charged patches in the moose Chn are similar to those in the bovine Chn, but patch 2 has intermediate characteristics between the corresponding patches in the bovine and camel Chn. An additional patch 4 in the moose Chn is located close to the substratebinding cleft (see Fig. 2). It is challenging to conclude whether the differences in positively charged patches in chymosins are stochastic or whether they result from adaptation to some conditions, such as species-specific variations in κ-caseins charge distributions. Further studies of the chymosins from different mammals may clarify this question.

The resulting preparation of rChn-Alc is able to coagulate cow’s milk. In terms of specific MA, however, it is inferior to the reference commercial rChns of cow and camel. It means that in cheese-making the consumption of the rChn of moose, obtained in the E. coli expression system, will be higher than that of rChn-Bos and rChn-Cam. In order to compete with reference enzymes, the specific MA of the moose rChn should be increased 3–4 times. However, in a number of technological parameters, the moose rСhn is superior to the reference commercial enzymes. Thus, in comparison with the rChn of a cow and a camel, the specific activity of the rChn of moose is less dependent on changes in the concentration of CaCl2 in the range of 1–5 mM and pH in the range of 6–7, which is an attractive technological property.

In general, though obtained in the prokaryotic system, the moose chymosin meets the basic requirements for enzymes for cheese-making, encouraging us to study this protein. The main problem of yeast expression systems is a strong ability to glycosylate proteins. Pichia may have an advantage in the glycosylation of secreted proteins over Saccharomyces cerevisiae because the former does create proteins with long carbohydrate chains via hyperglycosylation (Akishev et al., 2021). In an experiment to obtain recombinant camel chymosin, the prochymosin gene was successfully cloned and expressed in P. pastoris under the control of the GAP promoter and purified from culture via a combination of cation and anion exchange chromatography. Camelus bactrianus recombinant chymosin manifested high milk-clotting activity (9605 U/mg) (Akishev et al., 2021). One of the priority tasks is to obtain the moose rChn in the eukaryotic expression system and to compare its technological properties (primarily specific MA) with the properties of the enzyme produced in the E. coli expression system.

## Conclusion

The nucleotide sequence encoding moose (Alces alces) prochymosin was optimized for its efficient expression in E. coli cells of the SHuffle express strain. The synthesized prochymosin gene was cloned into the pET21a vector, resulting in the pET21-CYM-Alc expression vector. The constructed model of the spatial structure of the moose Chn showed that the ionic charges on the surface of the protein molecule are distributed similarly to those for the cow and camel Chn, but the moose enzyme has a unique charged site, which probably affects its MA.

A sample of moose rProChn was developed and its biochemical and technological properties were studied. In some of the technological parameters, it surpasses the reference commercial enzymes. Thus, the specific activity of the moose rChn is less dependent on changes in CaCl2 concentration in the range of 1–5 mM and substrate pH in the range of 6–7, compared to the cow and camel rChn. The total proteolytic activity of the moose rChn occupies an intermediate position between the cow and camel rChn. In terms of such indicators as specific milk-clotting activity, specificity and thermal stability, the moose rChn is inferior to reference commercial chymosins.

## Conflict of interest

The authors declare no conflict of interest.
